# White matter hyperintensity reduction and outcomes after minor stroke

**DOI:** 10.1212/WNL.0000000000004328

**Published:** 2017-09-05

**Authors:** Joanna M. Wardlaw, Francesca M. Chappell, Maria del Carmen Valdés Hernández, Stephen D.J. Makin, Julie Staals, Kirsten Shuler, Michael J. Thrippleton, Paul A. Armitage, Susana Muñoz-Maniega, Anna K. Heye, Eleni Sakka, Martin S. Dennis

**Affiliations:** From the Centre for Clinical Brain Sciences (J.M.W., F.M.C., M.d.C.V.H., K.S., M.J.T., S.M.-M., A.K.H., E.S., M.S.D.), UK Dementia Research Institute at the University of Edinburgh (J.M.W.), Centre for Cognitive Aging and Cognitive Epidemiology (J.M.W., M.d.C.V.H., S.M.-M.), University of Edinburgh; Institute of Cardiovascular and Medical Sciences (S.D.J.M.), University of Glasgow, UK; Department of Neurology and Cardiovascular Research Institute Maastricht (J.S.), Maastricht University Medical Centre, the Netherlands; and Academic Unit of Radiology (P.A.A.), Department of Cardiovascular Science, University of Sheffield, UK.

## Abstract

**Objective:**

To assess factors associated with white matter hyperintensity (WMH) change in a large cohort after observing obvious WMH shrinkage 1 year after minor stroke in several participants in a longitudinal study.

**Methods:**

We recruited participants with minor ischemic stroke and performed clinical assessments and brain MRI. At 1 year, we assessed recurrent cerebrovascular events and dependency and repeated the MRI. We assessed change in WMH volume from baseline to 1 year (normalized to percent intracranial volume [ICV]) and associations with baseline variables, clinical outcomes, and imaging parameters using multivariable analysis of covariance, model of changes, and multinomial logistic regression.

**Results:**

Among 190 participants (mean age 65.3 years, range 34.3–96.9 years, 112 [59%] male), WMH decreased in 71 participants by 1 year. At baseline, participants whose WMH decreased had similar WMH volumes but higher blood pressure (*p* = 0.0064) compared with participants whose WMH increased. At 1 year, participants with WMH decrease (expressed as percent ICV) had larger reductions in blood pressure (β = 0.0053, 95% confidence interval [CI] 0.00099–0.0097 fewer WMH per 1–mm Hg decrease, *p* = 0.017) and in mean diffusivity in normal-appearing white matter (β = 0.075, 95% CI 0.0025–0.15 fewer WMH per 1-unit mean diffusivity decrease, *p* = 0.043) than participants with WMH increase; those with WMH increase experienced more recurrent cerebrovascular events (32%, vs 16% with WMH decrease, β = 0.27, 95% CI 0.047–0.50 more WMH per event, *p* = 0.018).

**Conclusions:**

Some WMH may regress after minor stroke, with potentially better clinical and brain tissue outcomes. The role of risk factor control requires verification. Interstitial fluid alterations may account for some WMH reversibility, offering potential intervention targets.

White matter hyperintensities (WMH) are a common feature of cerebral small vessel disease (SVD) on brain MRI.^1^ Among several adverse clinical effects, WMH are associated with worsening cognition, double the risk of dementia, and triple the risk of stroke.^2^ A high WMH burden, vascular risk factors (particularly hypertension), and increasing age are associated with WMH,^3^ but other factors that influence WMH formation or longitudinal change remain poorly understood.

Most longitudinal studies focused on WMH progression, which, in population studies, usually occurs gradually.^3^ WMH reduction was reported in only 1 case report^4^ and in 14% of patients with stroke in a small case series,^5^ but those studies did not comment on any clinical effect or contemporaneous changes on other MRI parameters to provide confidence that the WMH reduction was not artifactual.

In a prospective study of clinical and imaging outcomes at 1 year after minor ischemic stroke,^6^ we unexpectedly observed visible reductions in the extent of WMH in some participants (figure 1). Therefore, here, we analyzed the proportions of participants with an increase, no change, or a decrease in WMH and determined whether WMH change was associated with recurrent cerebrovascular events, functional outcome, or changes in other imaging parameters.

**Figure 1:**
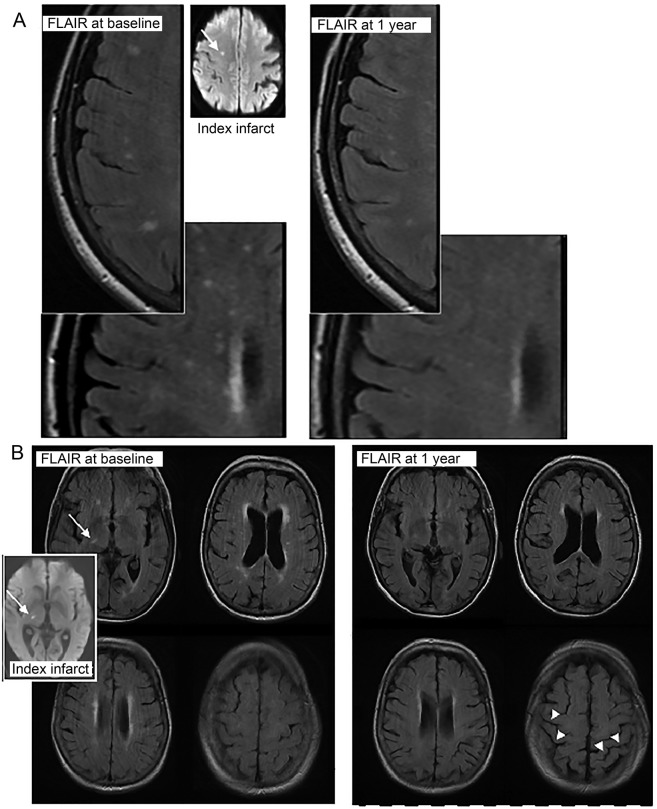
Examples of WMH reduction in 2 participants MRIs from patients (A, top and B, bottom) who showed definite visible reduction in WMH on MRIs between presentation with minor stroke (left, baseline; inset, the acute index infarct [arrow] on MRI diffusion tensor imaging) and 1 year (right). Note also the increase in visibility of sulci at 1 year (arrowheads, B, bottom right), indicating a reduction in brain volume accompanying the reduction in WMH volume. FLAIR = fluid-attenuated inversion recovery; WMH = white matter hyperintensities.

## METHODS

### Study design, participants, classification.

We recruited, prospectively and as consecutively as possible, patients who presented within 4 weeks of minor ischemic stroke of lacunar or cortical subtype to our regional stroke service between May 1, 2010, and May 31, 2012, as described previously.^6,7^ The regional stroke service is provided by a team of dedicated stroke physicians and specialists covering inpatients and outpatients, acute treatment, and secondary prevention. We focused the present analysis on participants who had brain MRI at presentation and at 1 year after stroke to assess WMH change. We included participants ≥18 years of age who were able to provide consent and excluded patients with moderate to severe stroke (NIH Stroke Scale [NIHSS] score >7), MRI contraindications, and hemorrhagic stroke.

All participants were assessed by a specialist stroke physician at presentation as part of their clinical stroke management and at recruitment to the study for research purposes. We recorded symptoms, medical history, vascular risk factors (diagnosis of hypertension, diabetes mellitus, hyperlipidemia, smoking status, blood pressure [BP]), medications, and alcohol use, and we performed a full clinical examination. We assessed NIHSS, BP (sitting, automated cuff, Omron Corp, Kyoto, Japan), and carotid stenosis (color Doppler ultrasound imaging) and performed ECG and blood and urine analyses.^7^

All participants underwent structural and brain diffusion tensor imaging (DTI) at presentation to confirm the acute index infarct. Participants with clinically definite stroke but no DTI-visible lesion were included if no alternative explanation for stroke symptoms was found. We assigned a definitive stroke subtype (lacunar or cortical) using the Oxfordshire Community Stroke Project Classification clinical syndrome^8^ and MRI DTI findings (recent small subcortical or cortical infarct).^9^ If no DTI lesion was visible (as occurs in 30% of minor strokes^10^), the clinical subtype^8^ was used.

All participants received treatment according to UK guidelines: antiplatelet drugs (mostly CLOPIDOGREL), a statin (mostly simvastatin), antihypertensive drugs (target 130 mm Hg systolic), and oral anticoagulants for atrial fibrillation. In addition, they were encouraged to stop smoking, to exercise, to reduce their salt intake, and to watch their weight.

### Follow-up.

We assessed all participants at 1 year after stroke, in person, for dependency (modified Rankin Scale [mRS] using a structured assessment tool), recurrent stroke or TIA, vascular risk factors, and BP, and we repeated the MRI. All clinical assessments were masked to imaging and vice versa.

### MRI studies.

All MRI examinations were performed on the same MRI scanner (1.5T Signa HDxt, General Electric, Milwaukee, WI) with self-shielding gradients and an 8-channel phased-array head coil. MRI included DTI, fluid-attenuated inversion recovery, T2-weighted, T2*- and T1-weighted volume sequence (published protocol,^7^ table e-1 at Neurology.org). Daily quality assurance tests were performed to maintain scanner uniformity.

### Image analysis.

#### Visual.

We defined the infarct responsible for the presenting symptoms as index, old infarcts present at baseline as old, and infarcts occurring between presentation and 1 year as incident. Index infarcts were identified as hyperintense on DTI/hypointense on apparent diffusion coefficient maps and/or hyperintense on fluid-attenuated inversion recovery, T2-weighted, and hypointense on T1-weighted images, possibly with minor mass effect but no ex vacuo effect.^9^ Small subcortical infarcts^9^ were rounded or ovoid, <2 cm in diameter in subcortical gray or white matter. Cortical infarcts involved cortex.^8^ Older infarcts were identified by shape, ex vacuo effect, and typical malacic signal characteristics. We categorized WMH using the Fazekas score.^11^ We assessed 1-year imaging first blinded to baseline and then comparing 1-year imaging with baseline to identify incident infarcts and hemorrhages and to score WMH change visually using the Prins WMH Change score.^12^

#### Computational.

We registered all structural images to the T1 sequence (http://fsl.fmrib.ox.ac.uk/fsl/fslwiki/FLIRT).^13^ An experienced analyst, blinded to clinical details, used a validated multispectral method (www.sourceforge.net/projects/bric1936) to measure intracranial volume (ICV) and whole-brain and WMH volumes and separated index/old/incident infarcts from WMH manually to avoid errors in WMH volume.^14^ We standardized brain and WMH volumes to ICV (expressed as percentage of ICV). We registered WMH maps to DTI images nonlinearly^15^ to measure mean diffusivity (MD) and fractional anisotropy in WMH and normal-appearing white matter. By the above method, the mean difference in WMH volume on repeated measures was 0.712 mL (95% CI −4.451 to 3.028) for a mean WMH volume of 21.96 ± 24.84 mL.

### Statistical analysis.

We assessed differences between participants with MRI at baseline and 1 year (the present analysis) and those who did not have MRI at 1 year.

We combined deep and periventricular Fazekas scores into a total WMH score (0–6). We combined clinically evident recurrent stroke or TIA and incident infarct on imaging into a composite outcome of any recurrent cerebrovascular event. We compared change in WMH by volume and visual scores and tested WMH volume change for regression to the mean. For display purposes and data summaries (but not statistical modeling), we divided WMH volume change into quintiles (Qs), from greatest reduction (Q1) to greatest increase (Q5).

We used analysis of covariance (ANCOVA) to assess predictors of follow-up WMH volume, including baseline vascular risk factors. We adjusted for baseline WMH volume using the cube root of the WMH volume normalized to ICV (i.e., percent ICV) because this improved model fit. We also used a model of changes^16,17^ in which the outcome variable is the difference in WMH volume between baseline and 1 year and predictors are differences in other variables, e.g., mean arterial BP. We used multinomial logistic regression to assess the relationship between 1-year mRS score and WMH change. We tested whether WMH change related to change in brain volume, baseline MD, or MD change at 1 year and index stroke visible/not visible on DTI. WMH change modeling retained direction of change (negative or positive).

All available data were used in all analyses. Predictor variables with strong relationships with other variables were removed to minimize collinearity (estimated with variance inflation factors). We restricted predictor variables to 1 per 10 observations.^18^ We did not impute data. The *p* values are 2 sided.

### Standard protocol approvals, registrations, and patient consents.

The study was approved by the Lothian Ethics of Medical Research Committee (REC 09/81101/54) and NHS Lothian R+D Office (2009/W/NEU/14). All participants gave written informed consent.

## RESULTS

Of 264 participants recruited, 190 had MRI at baseline and 1 year and are the subject of the current analysis. Of the 74 of 264 participants not included in the present analysis, follow-up MRI was not possible in 61 (22 were too unwell, 5 died, 34 declined) and was incomplete in 8, and 5 participants were not contactable (in all, 259 of 264 [98%] were followed up). The 74 participants who did not have MRI at 1 year were older but did not differ in proportions with vascular risk factors, stroke subtype, or baseline WMH volume from the 190 participants with 1-year MRI.

The 190 included participants had a mean age of 65.3 years (SD ± 11.3 years, range 34.3–96.9 years); 112 (59%) were male; and 87 (46%) had lacunar ischemic stroke (table 1). The median time between stroke and MRI was 4 days (interquartile range [IQR] 2–10 days); median NIHSS score was 1 (range 0–7); 139 of 190 (73%) had hypertension; mean arterial BP was 102.8 ± 15.4; 77 of 190 (41%) were current/recent smokers; 15 of 190 (8%) had atrial fibrillation; and 6 of 190 (3%) had 70% to 99% ipsilateral carotid stenosis.

**Table 1:**
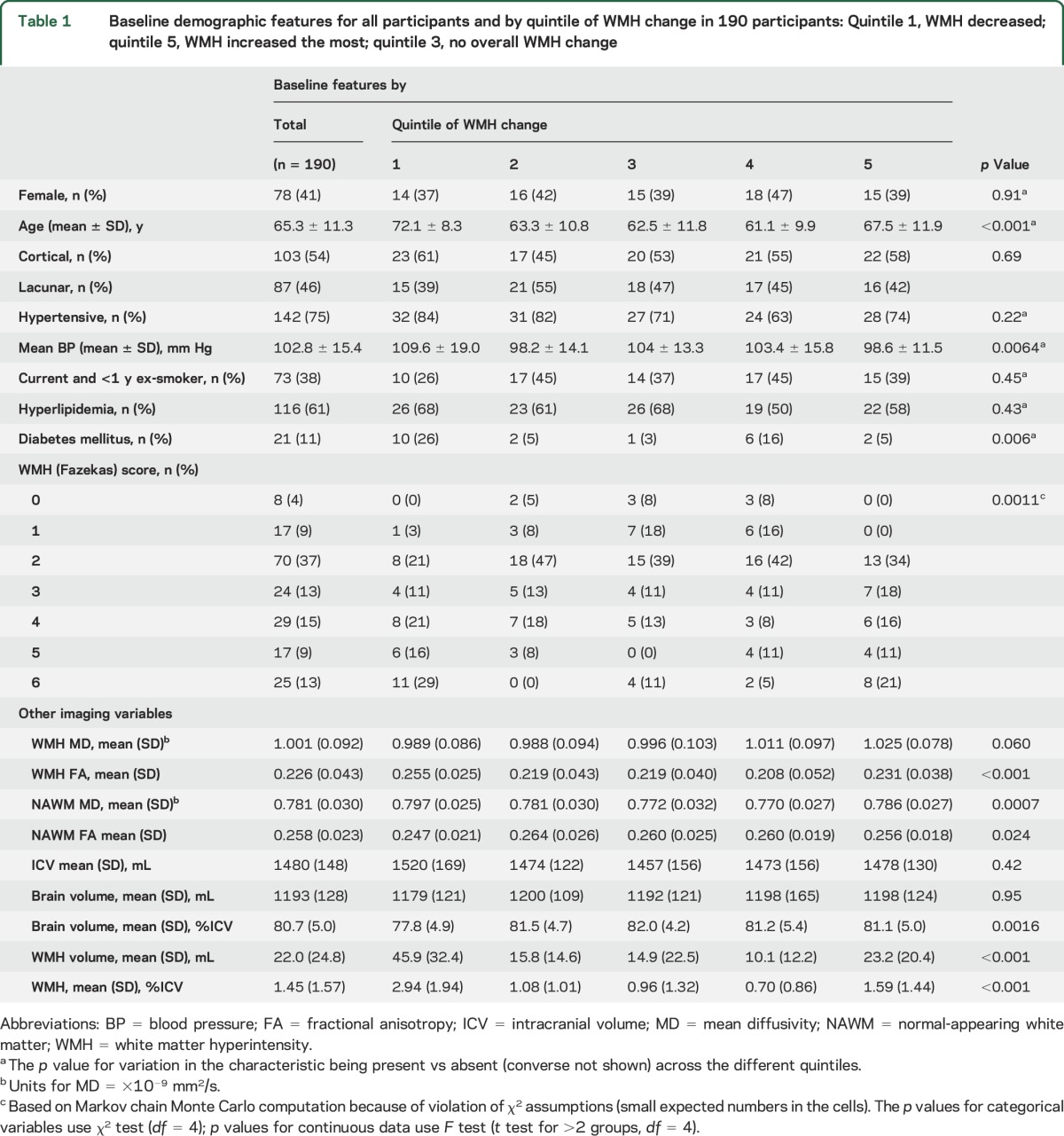
Baseline demographic features for all participants and by quintile of WMH change in 190 participants: Quintile 1, WMH decreased; quintile 5, WMH increased the most; quintile 3, no overall WMH change

The mean baseline WMH volume was 21.96 ± 24.84 mL (minimum 0 mL, maximum 121.48 mL, or 1.45 ± 1.57% of ICV); median WMH volume was 12.56 mL (IQR 4.00–32.70 mL); and Fazekas score was 2.5 of 6 (table 1). The mean WMH volume at 1 year was 23.23 ± 23.28 mL (minimum 0.8 mL, maximum 121.56 mL), with a mean change of 1.27 ± 8.46 mL overall (median WMH volume 14.97 mL [IQR, 5.86–32.74 mL], median change 1.42 mL [IQR −2.33 to 4.58 mL]). In multivariable modeling to predict WMH volume at 1 year, without considering change in WMH from baseline, participants with the most WMH at baseline had the most WMH growth at 1 year (β = 0.854 per baseline WMH as percent ICV, 95% CI 0.781–0.927, *p* < 0.0001, ANCOVA). Baseline WMH MD (β = 0.077 per 1-unit difference in baseline MD, 95% CI 0.022–0.133, *p* = 0.007) and baseline mean BP (β = −0.0006 per 1-mm Hg higher baseline arterial pressure, 95% CI −0.00099 to −0.00017, *p* = 0.005) were also significant predictors of WMH volume at 1 year, which was inverse for BP.

**Figure 2:**
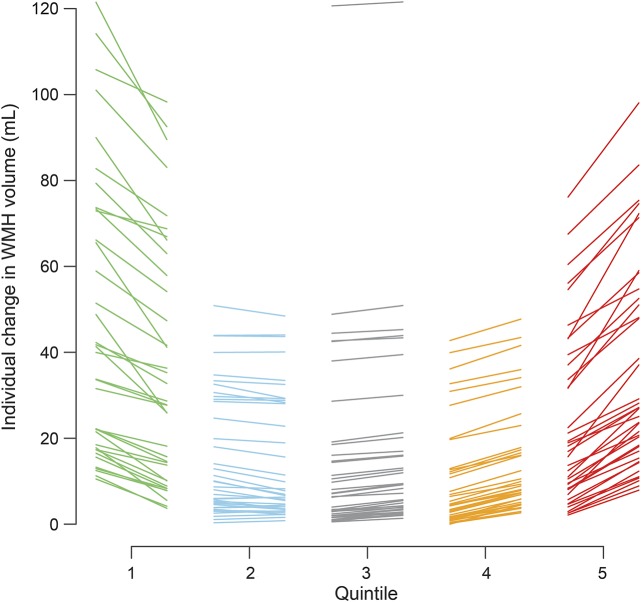
Individual participants' changes in WMH volume between baseline and 1 year by quintile of WMH volume change Each line represents an individual patient, linking baseline WMH volume (left) to follow-up WMH volume (right) of each quintile column. WMH = white matter hyperintensities.

**Figure 3:**
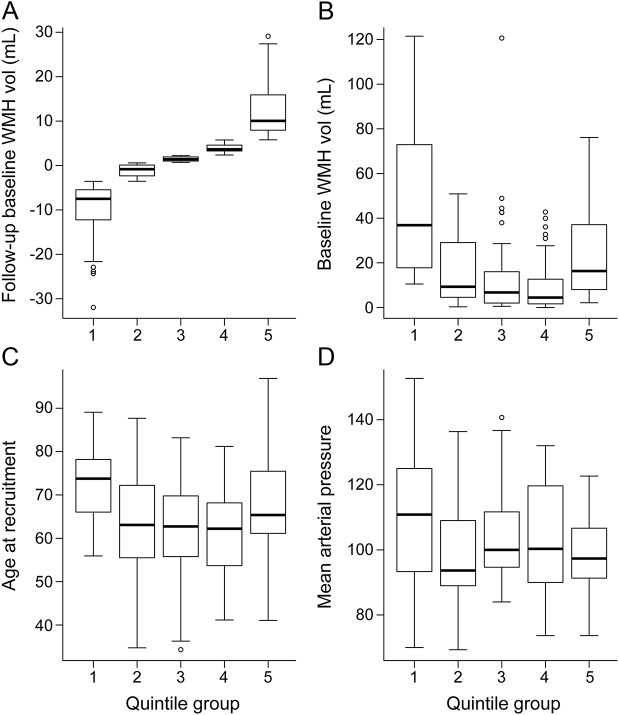
WMH change according to (A) WMH volume at 1 year, (B) WMH volume at baseline, (C) patient age, and (D) mean arterial blood pressure at baseline Participants in Q1 had the most WMH shrinkage and those in Q5 had the most WMH growth between baseline and 1 year. In the box and whiskers, the central line is median, box lower margin is 25th percentile, upper margin is 75th percentile, lower whisker is 5th percentile, and upper whisker is 95th percentile. WMH vol = white matter hyperintensity volume.

WMH volume decreased by some degree in 71 of 190 participants (table 1). For display purposes, we plotted WMH change in quintiles, from the greatest WMH reduction in Q1 to the greatest increase in Q5 (figure 2). This revealed that although there was a wide range of baseline WMH volumes in each change quintile, in general, there was a U-shaped relationship between baseline WMH and WMH change, with the highest baseline WMH volumes being in Q1 and Q5 and the lowest in Q3 and Q4 (*p* < 0.001, *F* test, figure 3). The ranges of change in WMH were −31.97 to −3.57 mL for Q1, −3.55 to 0.59 mL for Q2, 0.71 to 2.21 mL for Q3, 2.36 to 5.76 mL for Q4, and 5.78 to 29.11 mL for Q5. However, on average, the largest increases and decreases in WMH volumes occurred in the middle range of baseline WMH values, i.e., ≈40 mL (figure e-1), providing little evidence of regression to the mean. Age also showed a U-shaped relationship with WMH volume change, the oldest participants being in Q1 and Q5 (*p* < 0.001, *F* test, figure 3C and table 1). Mean baseline BP was highest in Q1 and lower in Q5 (*p* = 0.0064, *F* test, figure 3D). Many of the 21 participants with diabetes mellitus were in Q1 (*p* = 0.006). There were equal proportions with a diagnosis of hypertension, with hyperlipidemia, who smoked, and by stroke subtype per quintile.

Larger falls in mean arterial BP between baseline and 1 year were associated with more WMH reduction (adjusted β = 0.0053 percent ICV per 1 mm Hg, 95% CI 0.00099–0.0097, *p* = 0.017, table 2); a similar pattern was seen for systolic and diastolic BPs (figure e-2). There was no difference in prescribed antihypertensive drug classes between WMH quintiles (table e-2). There was no detectable association with change in smoking habit.

**Table 2:**
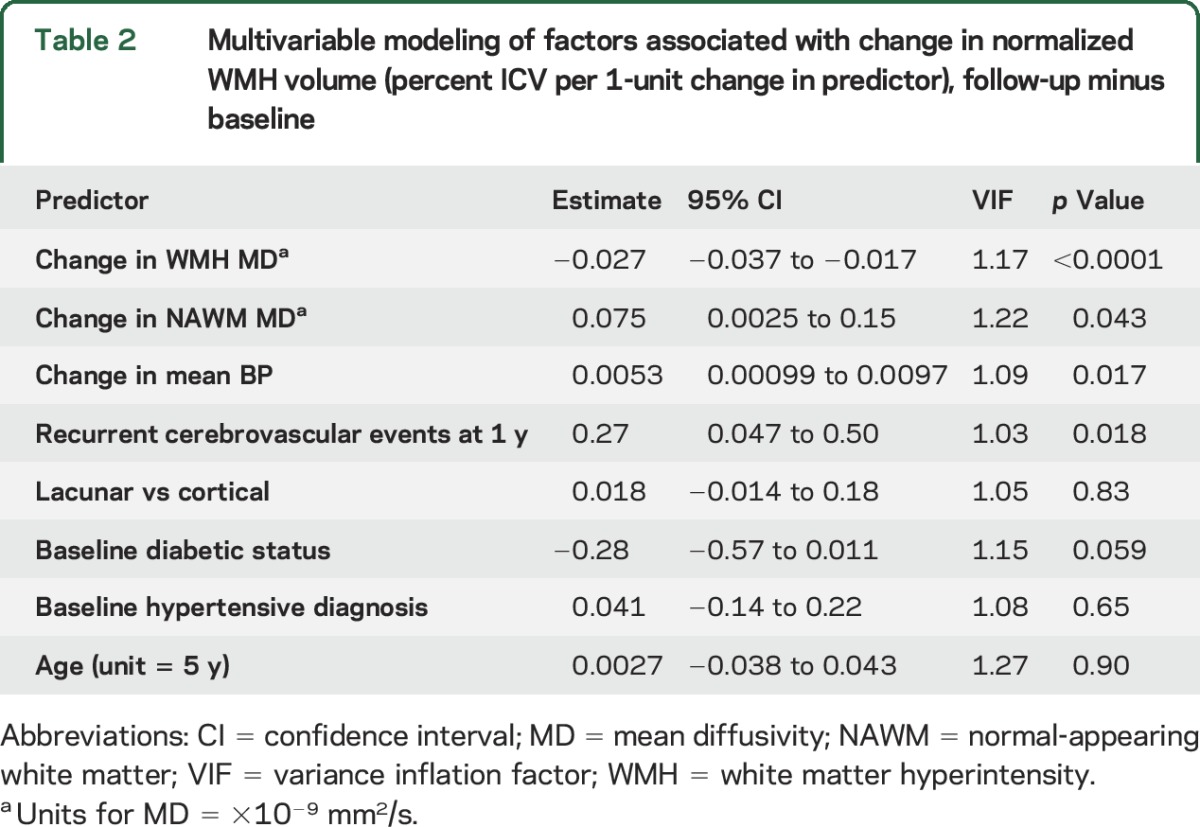
Multivariable modeling of factors associated with change in normalized WMH volume (percent ICV per 1-unit change in predictor), follow-up minus baseline

At 1 year, 35 of 190 (18.4%) participants had any recurrent cerebrovascular event (table e-3), which was associated with WMH growth rather than reduction (adjusted β = 0.27 WMH percent ICV per outcome event, 95% CI 0.047–0.50, *p* = 0.018, table 2). Most participants were independent (167 of 190 [87.9%]) with no difference in mRS scores by WMH change on univariate (*p* = 0.99, table e-3) or in adjusted analysis (table e-4).

To determine whether the WMH change was reflected in other tissue parameters (and therefore likely to be real), we assessed associations with MD, brain volume, visual WMH change scores, and visibility of the index infarct on DTI at presentation. In normal-appearing white matter, baseline MD and fractional anisotropy showed a U-shaped relationship with WMH change (*p* = 0.0007, table 1). Between baseline and 1 year, the MD changed significantly with WMH change in both WMH (β = −0.027 percent ICV, 95% CI −0.037 to −0.017, *p* < 0.0001) and normal-appearing white matter (β = 0.075 percent ICV, 95% CI 0.0025–0.15, *p* = 0.043, table 2). Brain volume appeared to decrease more in Q1 than in Q4 and Q5 between baseline and 1 year (figure e-3). Brain volume at 1 year was associated with WMH change when baseline brain volume was not considered (ANCOVA, β = 0.34, 95% CI 0.053–0.63, *p* = 0.020) but became nonsignificant when corrected for baseline brain volume (β = 0.14, 95% CI −0.098 to 0.38, *p* = 0.25), although the direction of effect remained the same. All reductions in WMH visual change scores were in Q1 through Q3, and most increases were in Q4 and Q5 (figure e-4). There was no association between visibility of the index infarct on DTI and WMH change (figure e-5).

## DISCUSSION

We demonstrate some reduction in WMH volume in participants presenting with minor ischemic stroke that was associated with fewer composite recurrent cerebrovascular events at 1 year and paralleled changes in other tissue parameters compared with participants with WMH growth. Thus, WMH may have some reversible component that may be clinically meaningful, supporting the concept that prevention of worsening WMH-related brain damage may translate into long-term benefits for brain health. While this observational study cannot identify causality, the association of WMH reduction with reduction in mean BP suggests that better risk factor management not only might attenuate WMH growth but also may actually reverse some WMH-related brain damage. The reduction in MD of normal-appearing white matter as WMH decreased could indicate improvements in white matter integrity that could limit progressive brain damage that leads to dementia.

Other factors, apart from decreasing BP, could have influenced WMH reduction, e.g., other secondary prevention (statins) or lifestyle improvements that participants might have adopted after the warning stroke (smoking cessation, exercise, diet). Although we did not find an effect of smoking cessation and were not able to assess changes in diabetes control, diet, or exercise, these interventions showed promise for reducing cognitive decline in community participants.^19^ Alternatively, while numerous observational studies show associations between high BP and WMH burden,^20,21^ randomized trials of BP reduction have produced mixed results. This might reflect that BP and hypertension account for only a small proportion of WMH variance^22^ or that arterial stiffness is key to WMH genesis rather than BP alone.^23^ There was no obvious difference in prescribed antihypertensive drug classes between the WMH change groups, and it would be inappropriate to interpret any class-quintile association as causative here. Future studies should assess the effects of drug class on WMH change and clinical outcomes.

WMH progression is related to baseline WMH load; therefore, participants in Q1 and Q2 should have had WMH progression similar to that in participants in Q4 and Q5. That the reduction in WMH volume is real, not artifact or regression to the mean (figure e-1), is supported by changes in MD, brain volume (figure 1 and figure e-3), and visual scores (figure e-4). The reduction in brain volume as WMH reduced is intriguing and suggests that interstitial fluid reduces from the (possibly increased) state at presentation with stroke, consistent with decreasing MD of normal white matter (table 2). A similar pattern of increased tissue fluid with increased brain volume early in WMH development was described in monogenic SVD.^24^

Most information on WMH longitudinal change comes from community-based, relatively stable participants, not patients with recent stroke whose brain changes may be more dynamic. Most longitudinal studies report no change or progression of WMH,^3^ but 4 community-based studies classified minor WMH regression as no progression^20,21^ or measurement error^3^ or did not discuss it.^12^ WMH regression was noted in 1 case report^4^ and 14 of 100 (14%) patients with stroke.^5^ We used a very sensitive, well-validated WMH volume method,^7^ as used in several recent stroke or aging studies totaling several thousand patients. We were careful to exclude all infarcts from the WMH volume.^14^ That, plus use of a careful quality-controlled magnetic resonance scanner, may have increased the sensitivity to detect bidirectional WMH changes. Future studies should assess tissue-level changes in WMH that regress or grow to determine how to identify lesions that are potentially reversible and scan more frequently to determine the timing and rate of change more precisely.

The study limitations include the complexities of differentiating WMH volume from infarcts.^14^ We manually differentiated index, old, and incident cortical and subcortical infarcts, checked volumes, and blinded all analyses. We cannot exclude that some WMH reduction reflects regression to the mean, but we do not think that all WMH changes (increases or decreases) can be attributed solely to this because both WMH increases and decreases occur along the full range of WMH baseline volumes (figure e-1). More scan-rescan studies are needed to determine WMH variability. We did not have ambulatory BP monitoring, but BP was measured carefully with validated instruments. We obtained follow-up on 259 of 264 participants (98.1%), but 34 of 264 declined repeat MRI (12.8%), which is low for a detailed MRI study in which frail older participants may need help attending hospital.

WMH pathogenesis is poorly understood. Lipohyalinosis, arteriolosclerosis, and fibrinoid necrosis describe perforating arteriolar wall damage, luminal narrowing, and occlusion that could cause chronic hypoperfusion and ischemia; blood-brain barrier (endothelial) failure may lead to perivascular edema and secondary brain damage.^1,6,25^ Multiple mechanisms may contribute to WMH, including augmentation. For example, leakage into the arteriolar wall may cause thickening, luminal narrowing, decreased vasoreactivity, and secondary ischemic damage superimposed on perivascular interstitial edema.^1^ The reduction in MD in normal-appearing white matter with WMH reduction suggests improved interstitial fluid balance. White matter microstructural damage precedes WMH appearance on MRI.^26^ Future studies should assess microstructure in regions of WMH growth and disappearance, with detailed cerebral and systemic vascular function measures, to identify mechanisms underlying WMH dynamics. WMH reversibility, not just absence of progression, has important consequences for identifying methods to prevent long-term SVD-related brain damage. WMH regression should be included in sample size calculations for trials using WMH as an intermediary endpoint. The possibility of minimizing deleterious clinical effects of WMH should encourage greater efforts to prevent vascular contributions to dementia.

## Supplementary Material

Data Supplement

Accompanying Editorial

## GLOSSARY

ANCOVA: analysis of covariance
BP: blood pressure
CI: confidence interval
DTI: diffusion tensor imaging
ICV: intracranial volume
IQR: interquartile range
MD: mean diffusivity
mRS: modified Rankin Scale
NIHSS: NIH Stroke Scale
Q: quintile
SVD: small vessel disease
WMH: white matter hyperintensities
