# Supplementary material for: White matter hyperintensity reduction and outcomes after minor stroke
Source: Neurology. 2017 Sep 5;89(10):1003–10. doi: 10.1212/WNL.0000000000004328 (PMC5589793; doi:10.1212/WNL.0000000000004328)
Supplement: Data Supplement [file supp_89_10_1003__index.html]

White matter hyperintensity reduction and outcomes after minor stroke — Data Supplement 

# White matter hyperintensity reduction and outcomes after minor stroke

## Data Supplement

**Neurology® data supplements are not copyedited before publication. Published editorials and translations have been copyedited.  
 © 2017 American Academy of Neurology.  
  
 Files in this Data Supplement:**

- Data Supplement - Microsoft Word file
