# Supplementary material for: White matter hyperintensity reduction and outcomes after minor stroke
Source: Neurology. 2017 Sep 5;89(10):1003–10. doi: 10.1212/WNL.0000000000004328 (PMC5589793; doi:10.1212/WNL.0000000000004328)
Supplement: Data Supplement [file supp_WNL.0000000000004328_Supplemental_Data.doc]

**Title: White matter hyperintensity reduction and outcome after minor stroke.**

**SUPPLEMENTARY FIGURES AND TABLES**

**Figure e-1** Test of WMH change as regression to the mean Page 2

**Figure e-2** Blood pressure at presentation (top) and one year (bottom): Page 3

left to right: systolic, diastolic, mean arterial pressure, pulse pressure.

**Figure e-3** Change in brain volume between presentation and one year by WMH quintile. Page 4

**Figure e-4** WMH volume change quintile and change in WMH by Page 5

brain region assessed visually using the Prins Change Score.

**Figure e-5.** WMH change from baseline to one year versus visibility Page 6

of the index infarct on DTI at presentation.

**Table e-1** Magnetic resonance imaging sequence parameters. Page 7

Table e-2 Antihypertensive drug classes by quintile of WMH volume change Page 8

**Table e-3** Outcomes at one year by WMH quintile of change Page 9

**Table e-4** Predictors of functional outcome (modified Rankin Scale) at Page 9

one year including WMH volume change.

**Figure e-1. Testing regression to the mean for change in WMH volume between presentation and one year.**

The graph suggests little regression to the mean. Increases and decreases in WMH volume (y-axis) are spread fairly equally across the range of baseline WMH volumes (x-axis). Although patients with small baseline WMH volumes tended to increase, patients with large baseline volumes did not tend to decrease, the biggest decreases and increases occurring in the middle range of baseline volumes. Where regression to the mean is a major contributor to change, patients with the smallest volumes would have the largest increases, and those with the largest volumes would have the largest decreases.


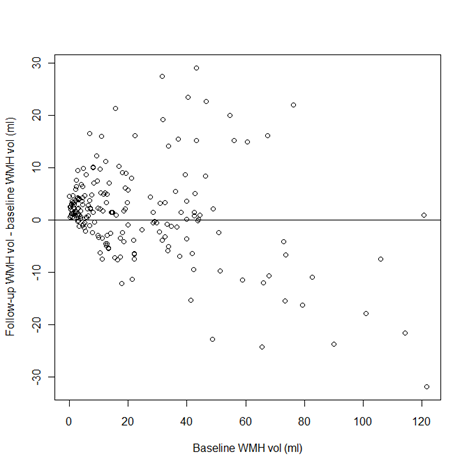


**Figure e-2. Blood pressures at presentation (top) and difference in BP between baseline and one year (bottom) by WMH change quintile**: left to right: systolic, diastolic, mean arterial pressure, pulse pressure.


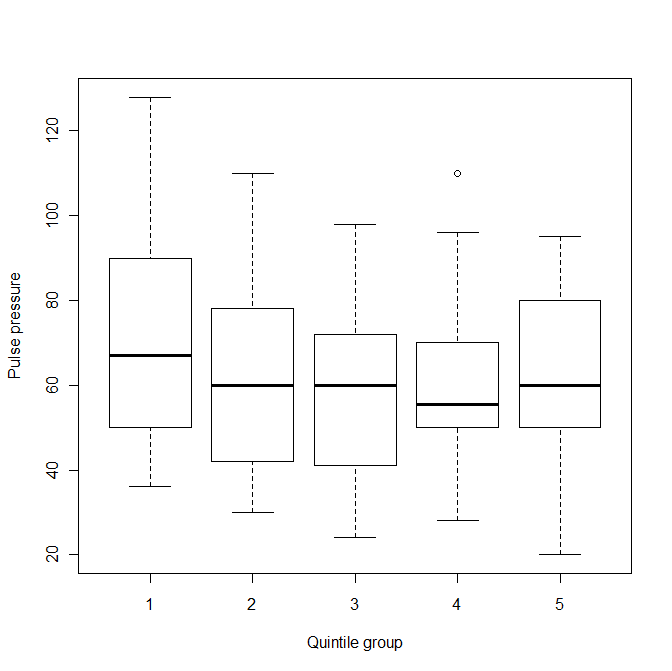

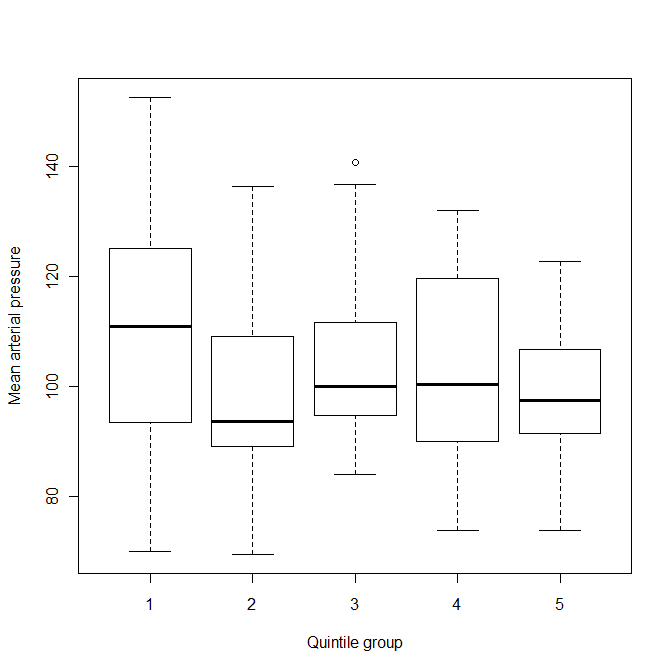

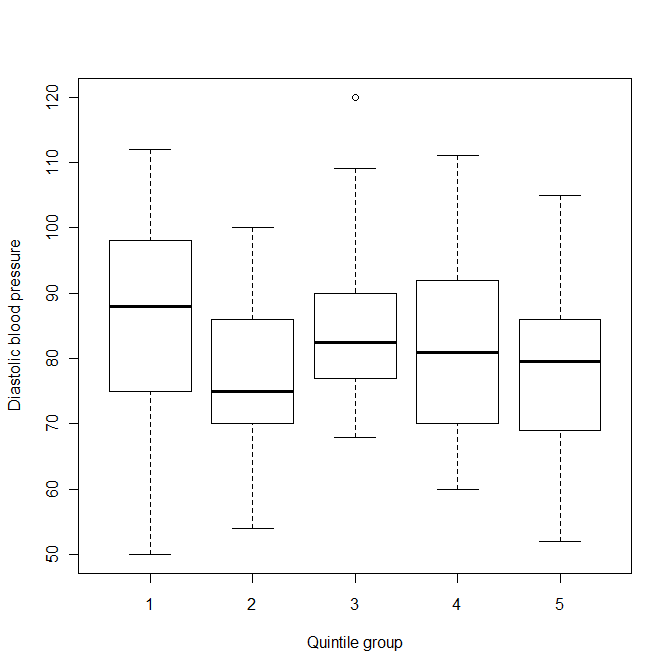

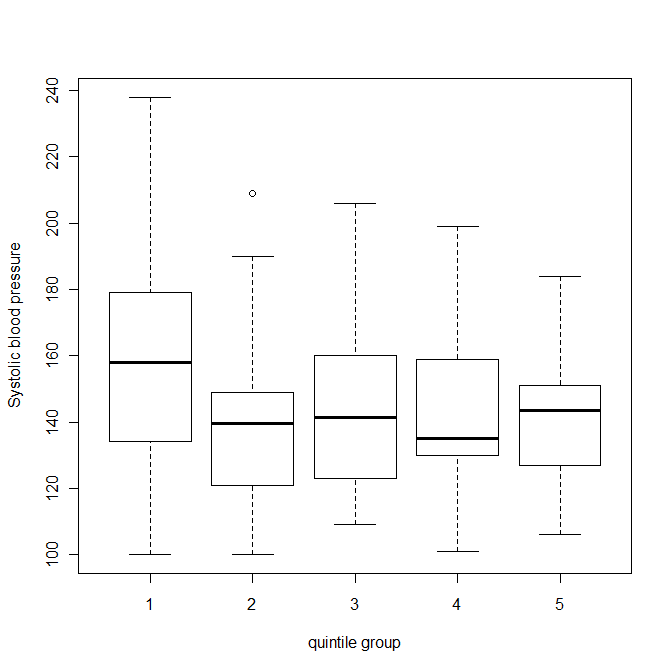

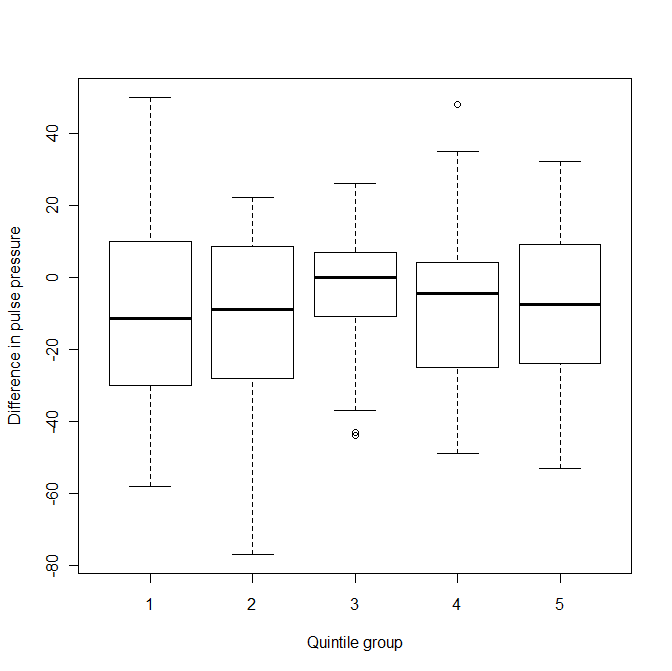

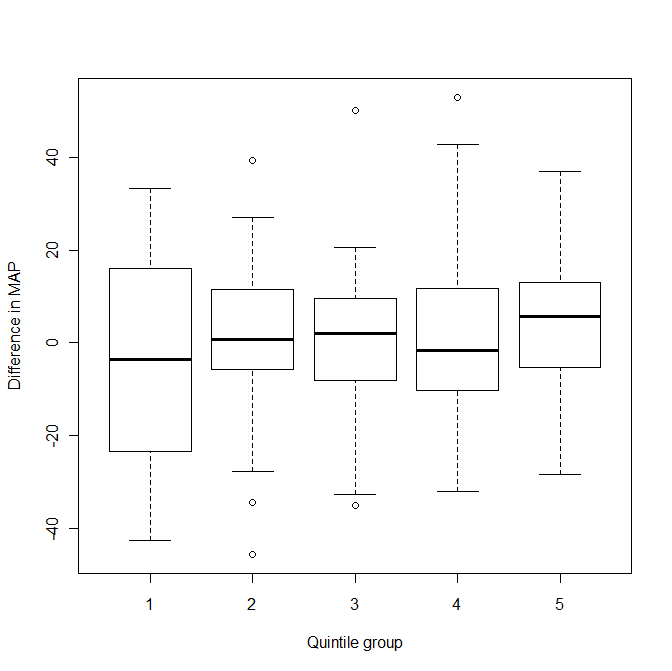

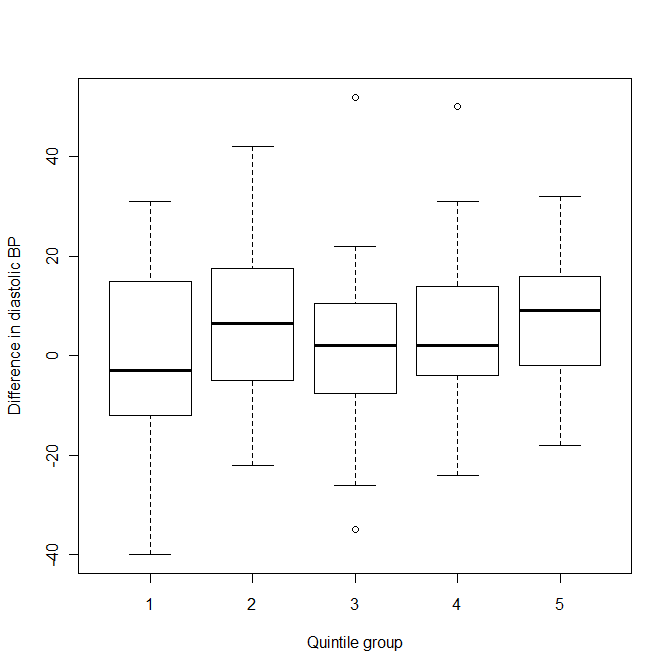

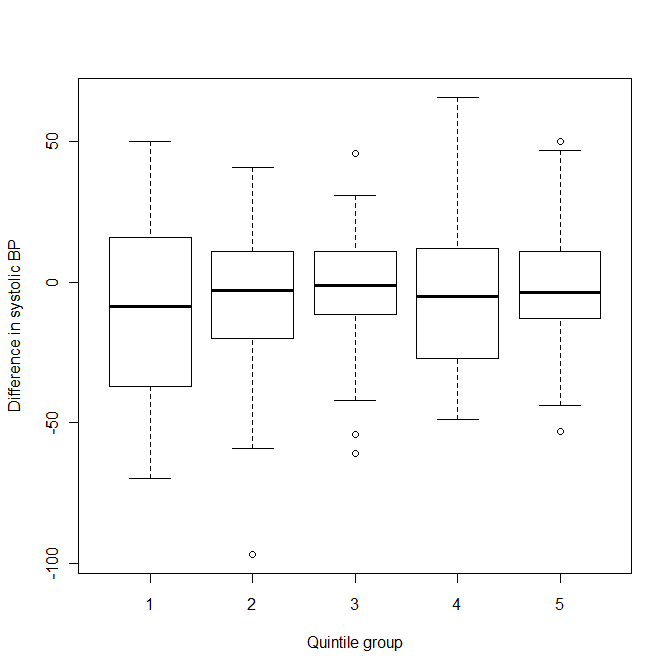


**Figure e-3** Change in brain volume between presentation and one year by WMH quintile.


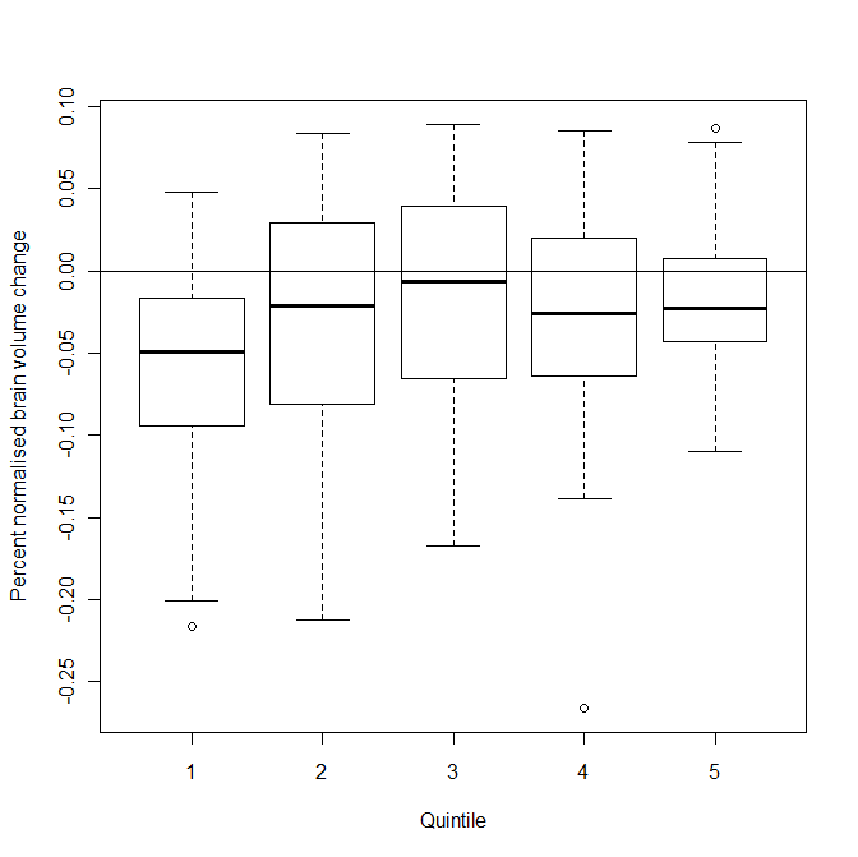


**Figure e-4. WMH volume change quintile and change in WMH by brain region assessed visually using the Prins Change Score.**


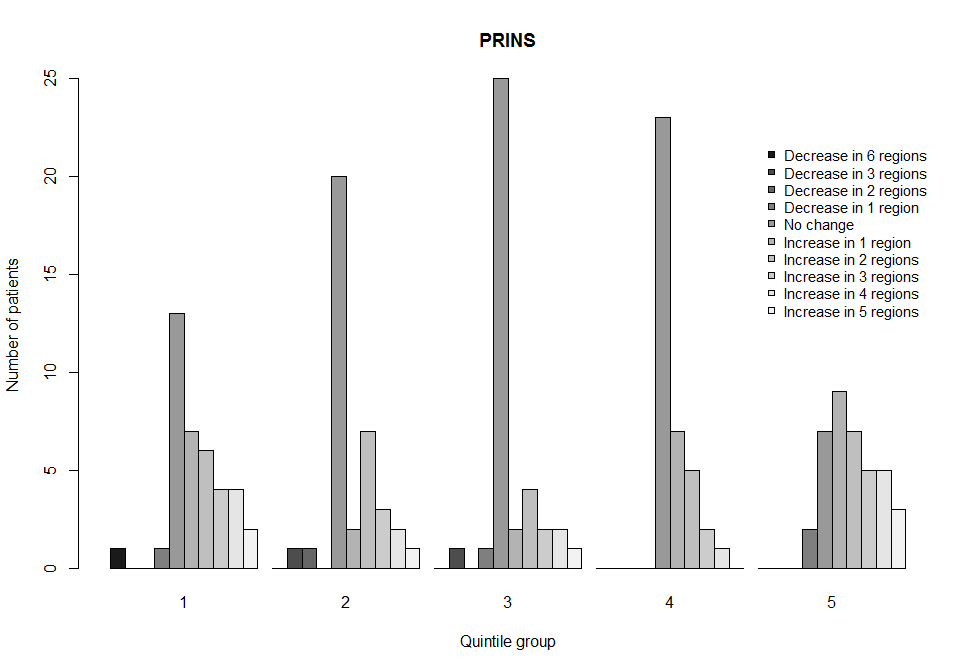


**Figure e-5. WMH change from baseline to one year versus visibility of the index infarct on DTI. There was no association between WMH shrinkage or growth and visibility on DTI of the index infarct. Top, WMH change by presence of DTI visible index infarct. Bottom, WMH change quintile and DTI visibility of the index infarct. The index infarct was visible on DTI in about 70% of patients.**


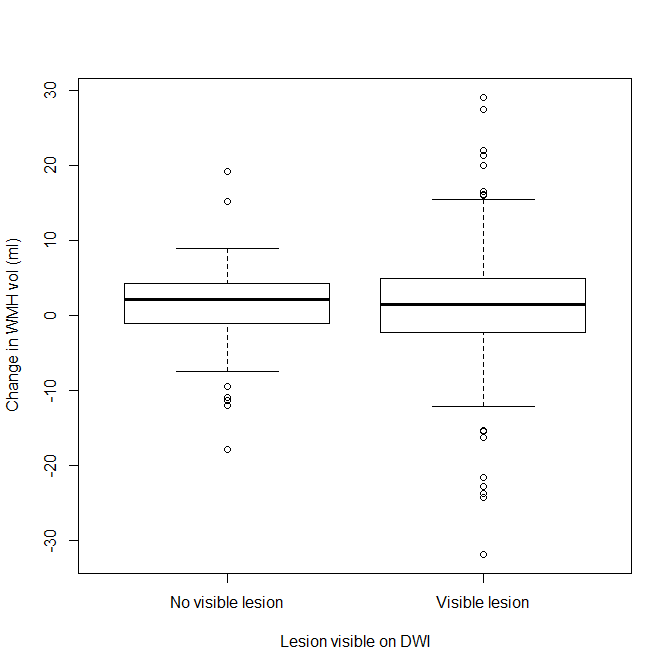


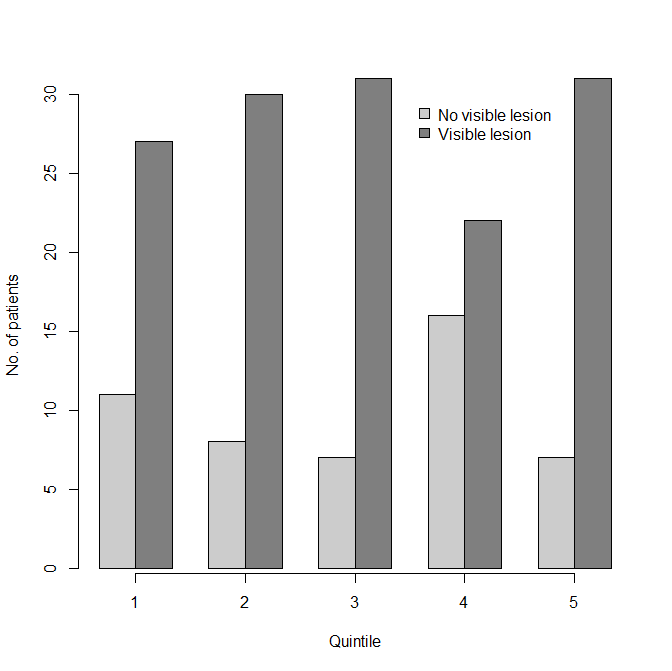


**Table e-1. Magnetic resonance imaging sequence parameters. The 1.5T GE Signa HDxt MR scanner had self-shielding gradients (33 mT/m) and 8-channel phased-array head coil. The same sequences were used at presentation and at one year follow-up.**

| **Sequence** | **T1W spin echo** | **DWI/DTI**  **(30 diffusion directions)** | **FLAIR (TI=2200 ms)** | **T2W Fast spin echo** | **T2*W gradient recalled echo (FA=20^o^)** | **3D IR PREP (TI=500ms, FA=8 ^o^)** | **SPGR**  **(FA=2 ^o^)** | **SPGR* (FA=12 ^o^)** |
| --- | --- | --- | --- | --- | --- | --- | --- | --- |
| Orientation | Sagittal | Axial | Axial | Axial | Axial | Sagittal | Axial | Axial |
| TE (ms) | 14 | 82 | 153 | 90 | 15 | 2.9 | 3.1 | 3.1 |
| TR (ms) | 400 | 7700 | 9000 | 6000 | 800 | 7.3 | 8.2 | 8.2 |
| FOV | 24 x 24 | 24 x 24 | 24 x 24 | 24 x 24 | 24(AP) x 18 | 330(SI) x 214.5 | 24 x 24 | 24 x 24 |
| Slice thickness (mm) | 5.0 | 5.0 | 5.0 | 5.0 | 5.0 | 1.8 | 4 | 4 |
| Slice gap (mm) | 0 | 1.0 | 1.0 | 1.0 | 1.0 | 0 | 0 | 0 |
| Matrix | 256(SI) x 160 | 128x128 | 384x224 | 384X384 | 384(AP)x168 | 256(SI)x146 | 256(AP)X192 | 256(AP)X192 |
|  |  |  |  |  |  |  |  |  |
| No. slices | 5 | 28 | 28 | 28 | 28 | 100 | 42 | 42 |
| Acquisition time | 0:54 | 4:14 | 4:48 | 2:30 | 4:32 | 4:17 | 1.13 | 1.13 |

^+^ sequence repeated after contrast injection 20 times consecutively.

Table e-2. Use of antihypertensive agent by quintile of WMH change. 38 patients per group. Some patients were taking more than one drug.

| **Drug** | **Q1** | **Q2** | **Q3** | **Q4** | **Q5** |
| --- | --- | --- | --- | --- | --- |
| Thiazide | 4 | 10 | 5 | 7 | 6 |
| Other diuretic | 1 | 0 | 2 | 0 | 5 |
| Alpha blocker | 2 | 0 | 2 | 2 | 3 |
| Beta blocker | 4 | 5 | 5 | 3 | 7 |
| AT2 Receptor Antagonist | 1 | 1 | 2 | 2 | 2 |
| ACE Inhibitor | 10 | 6 | 13 | 8 | 9 |
| Ca Channel Blocker | 3 | 9 | 4 | 6 | 8 |

Table e-3. Outcomes at one year (n, %) by WMH change quintile group, 1=most reduction, 5=most increase. (total n=190).

|  | **Total** | **Quintile of WMH change** | | | | | | | **p value** |
| --- | --- | --- | --- | --- | --- | --- | --- | --- | --- |
| **Neurological outcome** | N=190 | | 1 | 2 | 3 | 4 | 5 |  | |
| Recurrent stroke | 16 | | 3 | 3 | 2 | 3 | 5 |  | |
| % | 8.42 | | 1.58 | 1.58 | 1.05 | 1.58 | 2.63 |  | |
| Recurrent TIA | 5 | | 1 | 1 | 2 | 1 | 0 |  | |
| % | 2.63 | | 0.53 | 0.53 | 1.05 | 0.53 | 0 |  | |
| New lesion on scan | 20 | | 2 | 4 | 2 | 2 | 10 |  | |
| % | 10.58 | | 1.06 | 2.12 | 1.06 | 1.06 | 5.29 |  | |
|  |  | |  |  |  |  |  |  | |
| Any recurrent cerebrovascular event* | 35 | | 6 | 7 | 5 | 5 | 12 | 0.20 | |
| % | 18.42 | | 3.16 | 3.68 | 2.63 | 2.63 | 6.32 |  | |
| Free of any recurrent cerebrovascular event* | 155 | | 32 | 31 | 33 | 33 | 26 |  | |
| % | 81.58 | | 84.21 | 81.58 | 86.84 | 86.84 | 68.42 |  | |
|  |  | |  |  |  |  |  |  | |
| **Modified Rankin Scale** |  | |  |  |  |  |  |  | |
| mRS = 0 | 46 | | 8 | 9 | 11 | 9 | 9 | ^0.99 | |
| % |  | | 17.39 | 19.57 | 23.91 | 19.57 | 19.57 |  | |
| mRS = 1 | 67 | | 15 | 16 | 12 | 12 | 12 |  | |
| % |  | | 22.39 | 23.88 | 17.91 | 17.91 | 17.91 |  | |
| mRS = 2 | 54 | | 9 | 9 | 11 | 12 | 13 |  | |
| % |  | | 16.67 | 16.67 | 20.37 | 22.22 | 24.07 |  | |
| mRS = 3 | 23 | | 6 | 4 | 4 | 5 | 4 |  | |
| % |  | | 26.09 | 17.39 | 17.39 | 21.74 | 17.39 |  | |

*Any recurrent cerebrovascular event = either a recurrent stroke OR a TIA OR a new lesion on scan. ^Likelihood ratio chi-square = 3.48, df = 12

Table e-4. Predictors of functional outcome (modified Rankin Scale) at one year including WMH change. Proportional odds multinomial logistic regression. The AUC (area under curve) is an indication of how well the model predicts the mRS (0.586 indicates moderate prediction); the Brier is a measure of model performance (0.71 indicates rather moderate performance suggesting that important predictors are not accounted for in the model).

| **Odds Ratio Estimates (AUC = 0.586, Brier = 0.71)** | | | | |
| --- | --- | --- | --- | --- |
| **Effect** | **Point Estimate** | **95% Wald Confidence Limits** | | **P value** |
| Age (5 yr increments) | 1.025 | 0.901 | 1.166 | 0.71 |
| Baseline diabetes | 1.635 | 0.696 | 3.837 | 0.26 |
| Baseline hypertension | 1.167 | 0.613 | 2.222 | 0.64 |
| Baseline hyperlipidaemia | 1.227 | 0.702 | 2.146 | 0.47 |
| Baseline pulse pressure | 0.989 | 0.975 | 1.002 | 0.10 |
| Baseline smoker | 1.795 | 1.004 | 3.209 | 0.048 |
| Baseline stroke subtype | 0.992 | 0.589 | 1.673 | 0.98 |
| WMH volume difference (ml) | 0.990 | 0.960 | 1.022 | 0.55 |
