# Supplementary material for: White matter hyperintensity reduction and outcomes after minor stroke
Source: Neurology. 2017 Sep 5;89(10):1003–10. doi: 10.1212/WNL.0000000000004328 (PMC5589793; doi:10.1212/WNL.0000000000004328)
Supplement: Accompanying Editorial [file supp_WNL.0000000000004328_984.pdf]

Waxing and waning of white matter hyperintensities

Frank-Erik de Leeuw, MD, PhD
Fenwick Nichols III, MD

Correspondence to Dr. de Leeuw: FrankErik.deLeeuw@radboudumc.nl

Neurology® 2017;89:984–985

Small vessel disease (SVD) manifests in myriad ways, most prominently as white matter hyperintensities (WMH).[1,2] By the age of 60 years, virtually every healthy individual has evidence of WMH,[3] and those with ischemic stroke have even more extensive changes.[4] Cerebral ischemic injury from occlusion or stenosis of deep penetrating arteries due to (uncontrolled) hypertension ranks highest among the potential causes of WMH.[5] These WMH are among the most important vascular contributors to cognitive decline, dementia, and parkinsonism.[6]

Data from longitudinal studies show that both the prevalence and severity of WMH increases with age.[7] Reports of WMH regression have been suspected to reflect measurement error.[7] This has led to the generally accepted notion that the burden of WMH increases gradually over time, without formal evidence.

In this issue of *Neurology*®, Wardlaw et al.[1] investigate changes in volume of WMH over time in a systematic approach. They explicitly consider the possibility of reductions in volume, rather than just looking at increases. Surprisingly, they found that a substantial proportion, over one third of all patients, exhibited regression of WMH volumes 1 year after initial neuroimaging. These individuals also experienced a greater drop in mean arterial blood pressure over the course of 1 year, compared with those who experienced an increase in WMH volume. Decliners also had a lower risk of recurrent cerebrovascular events.

These results, together with the recognition of acute ischemia as an etiology of SVD,[8] reflect a rapidly evolving and dynamic SVD field as opposed to the traditional belief of chronic ischemia relentlessly leading to progression of WMH.[5]

However, several issues require further attention. Because the greatest reduction occurred in those patients with the highest baseline volumes of WMH, there is a need to address the potential for measurement error, due to scan variability or segmentation error. In addition, the individuals who experienced the greatest decrease in WMH volume (Q1) also had the smallest fraction of brain volume, relative to their intracranial volume. Although they did not report the rate of cerebral atrophy, brain atrophy could occur at a higher pace in patients with an already low brain volume (perhaps due to earlier brain atrophy) than in patients with a higher baseline brain volume (Q2-5), and the WMH volumes could atrophy as well.

Pathophysiologically, reduction in edema in the year after the qualifying stroke could explain the regression in WMH. However, how a cortical stroke, remote from the WMH, might result in WMH edema that ultimately resolves over the course of 1 year needs further investigation.

One final question remains when it comes to the external validity of these findings. The authors correctly point out that WMH are increasingly considered as the most important contributing factor to dementia,[9] but also of vascular parkinsonism.[6] However, these lesions are generally observed within the context of population-based studies or studies with patients with symptomatic SVD (e.g., lacunar stroke or vascular cognitive or motor impairment), rather than in the acute stroke context.[6]

The findings of Wardlaw et al. should therefore first be viewed as a keen but deviant observation of the communis opinio on how changes of WMH occur over time, and that observation warrants further investigation in the aforementioned patient groups with cerebral SVD. One strategy suggested by the authors would be using a higher imaging frequency to gain insight into the etiology of progression of WMH, and thereby of SVD over time.[8]

As a highly prevalent condition that causes numerous disabling disorders with high costs to society, WMH warrants proper investigation of its etiology. Regression of WMH might ultimately open completely new therapeutic strategies in the amelioration of these deleterious and costly consequences of cerebral SVD in general, and specifically those of WMH.

ACKNOWLEDGMENT

Prof. de Leeuw is supported by a clinical established investigator grant of the Dutch Heart Foundation (grant 2014 T060) and by a VIDI innovational grant from The Netherlands Organisation for Health Research and Development (ZonMw grant 016-126-351).

See page 1003

From the Department of Neurology (F.-E.d.L.), Donders Institute for Brain, Cognition and Behaviour, Center for Neuroscience, Radboud University, Nijmegen, the Netherlands; and Department of Neurology (F.N.), Medical College of Georgia at Augusta University.

Go to Neurology.org for full disclosures. Funding information and disclosures deemed relevant by the authors, if any, are provided at the end of the editorial.

 © 2017 American Academy of Neurology

© 2017 American Academy of Neurology. Unauthorized reproduction of this article is prohibited.

STUDY FUNDING

No targeted funding reported.

DISCLOSURE

The authors report no disclosures. Go to Neurology.org for full disclosures.

REFERENCES

1. Wardlaw JM, Chappell FM, Valdés Hernández MC, et al. White matter hyperintensity reduction and outcomes after minor stroke. Neurology 2017;89:1003–1010.
2. Wardlaw JM, Smith EE, Biessels GJ, et al. Neuroimaging standards for research into small vessel disease and its contribution to ageing and neurodegeneration: a united approach: standards for reporting vascular changes on neuroimaging. Lancet Neurol 2013;12:822–838.
3. de Leeuw FE, de Groot JC, Oudkerk M, et al. Prevalence of cerebral white matter lesions in elderly people: a population based magnetic resonance imaging study: The Rotterdam Scan Study. J Neurol Neurosurg Psychiatry 2001;70:9–14.
4. Fu JH, Lu CZ, Hong Z, Dong Q, Luo Y, Wong KS. Extent of white matter lesions is related to acute subcortical infarcts and predicts further stroke risk in patients with first ever ischaemic stroke. J Neurol Neurosurg Psychiatry 2005;76:793–796.
5. Pantoni L. Cerebral small vessel disease: from pathogenesis and clinical characteristics to therapeutic challenges. Lancet Neurol 2010;9:689–701.
6. van der Holst E, van Uden IWM, Tuladhar AM, et al. Cerebral small vessel disease and incident parkinsonism: the RUN DMC study. Neurology 2015;85:1569–1577.
7. Schmidt R, Seiler S, Loitfelder M. Longitudinal change of small-vessel disease-related brain abnormalities. J Cereb Blood Flow Metab 2016;36:26–39.
8. Lyoubi-Idrissi AL, Jouvent E, Poupon C, Chabriat H. Diffusion magnetic resonance imaging in cerebral small vessel disease. Rev Neurol 2017;173:201–210.
9. Vermeer SE, Prins ND, den Heijer T, Hofman A, Koudstaal PJ, Breteler MMB. Silent brain infarcts and the risk of dementia and cognitive decline. N Engl J Med 2003;348:1215–1222.

© 2017 American Academy of Neurology. Unauthorized reproduction of this article is prohibited.
